# Magnetoencephalographic Correlates of Perceptual State During Auditory Bistability

**DOI:** 10.1038/s41598-018-19287-0

**Published:** 2018-01-17

**Authors:** Robert D. Sanders, Joel S. Winston, Gareth R. Barnes, Geraint Rees

**Affiliations:** 1 Institute of Cognitive Neuroscience University College London, Alexandra House, 17-19 Queen Square, London, WC1N 3AR London, United Kingdom; 2 Department of Anesthesiology, University of Wisconsin, Madison, USA; 3 Wellcome Trust Centre for Neuroimaging, University College London, London, WC1N 3BG United Kingdom

## Abstract

Bistability occurs when two alternative percepts can be derived from the same physical stimulus. To identify the neural correlates of specific subjective experiences we used a bistable auditory stimulus and determined whether the two perceptual states could be distinguished electrophysiologically. Fourteen participants underwent magnetoencephalography while reporting their perceptual experience while listening to a continuous bistable stream of auditory tones. Participants reported bistability with a similar overall proportion of the two alternative percepts (52% vs 48%). At the individual level, sensor space electrophysiological discrimination between the percepts was possible in 9/14 participants with canonical variate analysis (CVA) or linear support vector machine (SVM) analysis over space and time dimensions. Classification was possible in 14/14 subjects with non-linear SVM. Similar effects were noted in an unconstrained source space CVA analysis (classifying 10/14 participants), linear SVM (classifying 9/14 subjects) and non-linear SVM (classifiying 13/14 participants). Source space analysis restricted to *a priori* ROIs showed discrimination was possible in the right and left auditory cortex with each classification approach but in the right intraparietal sulcus this was only apparent with non-linear SVM and only in a minority of particpants. Magnetoencephalography can be used to objectively classify auditory experiences from individual subjects.

## Introduction

Bistable perception is a widely-adopted model for studying the neural correlates of consciousness^1^. Occurring when two alternative experiences can be derived from the same physical stimulus, it dissociates the conscious experience of a stimulus from the physical characteristics of the stimulus itself^2^. This provides a route to identify the neurophysiological underpinnings of two related but distinct perceptual states. Bistable visual stimuli have been widely employed, for example in binocular rivalry, where two different images presented simultaneously to the two eyes compete for dominance in the conscious stream^3–6^. In the auditory domain, bistability can be achieved through an auditory streaming task where a sequence of tones (typically played to both ears simultaneously) can result in two different auditory percepts (derived from the same sequence of tones)^1,7,8^. While visual bistability has been the subject of many investigations, auditory bistability has been relatively under-studied (though see references^1,9–16^). Hence the electrophysiological and neural correlates of subjective experience in auditory bistability are relatively poorly defined.

In a seminal study, Gutschalk *et al*.^9^ demonstrated that electrophysiological differences at approximately 259–265 ms after the onset of the triplet were able to distinguish the two perceptual states using magentoencephalography (MEG) with MEG dipoles focused in the auditory cortex (with no further relevant sources identified)^9^. Subsequent EEG studies focused on the auditory cortex made similar observations^17–19^. These studies have been supported by a functional magnetic resonance imaging study showing differences in evoked BOLD responses in primary and secondary auditory cortex^20^. However a follow up intracranial electroencephalography study was unable to identify discriminatory electrophysiological correlates of the bistable perception from temporal cortex^21^. Hence, as highlighted recently by Gutschalk and Dykstra, there remains ambiguity over the neural correlates of auditory bistability based on the available MEG and fMRI data^1^. Indeed, using functional magnetic resonance imaging (fMRI), Cusack was able to show that activity in the intraparietal sulcus (IPS) could discriminate perception of the segregated streams of tones from the gallop rhythm^22^ which was subsequently replicated^20^. IPS activity may thus relate to selection of the individual perceptual experience^23^ as changes in auditory cortex BOLD signals correlate with perceptual switches in auditory streaming too.

Given data from visual bistability suggesting that several different cortical regions contribute to the perception of visual stimuli^23–25^ further data providing insights for the electrophysiological and neural correlates of subjective experience in auditory bistability are required. Here we report results from a MEG study of auditory bistability that has potential to inform on these correlates.

## Results

**Figure 1:**
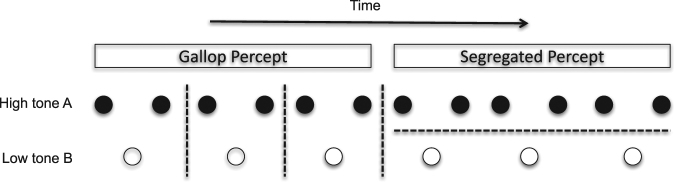
Schematic representing the two possible percepts from the bistable auditory stimulus of a triplet of tones. The tone length was 100 ms and within the triplet the inter-tone time was 50ms. Between each triplet of A-B-A there was a 200 ms gap.

Fourteen adults (aged between 18 to 51, mean = 24.5 years, standard deviation 8.3 years; six females) with unimpaired hearing gave informed consent to participate in the study which was approved by the UCL ethics committee. The behavioral task was modified from that previously employed by Gutschalk *et al*. whereby a triplet of tones (A-B-A) were played repeatedly whilst participants continuously reported their resulting conscious percept. The total duration of the triplet plus duration until the next triplet was 600ms (termed a trial). The A tone was 1414 Hz and the B tone was 1000 Hz. Typically, this stimulus results in two possible states described as a “gallop” rhythm (with each A-B-A triplet perceived as a unit) or a segregated stream of tones (with the percept of A-A-A and B-B as distinct streams). A schematic representing the differences in perception is included as Fig. 1.

**Figure 2:**
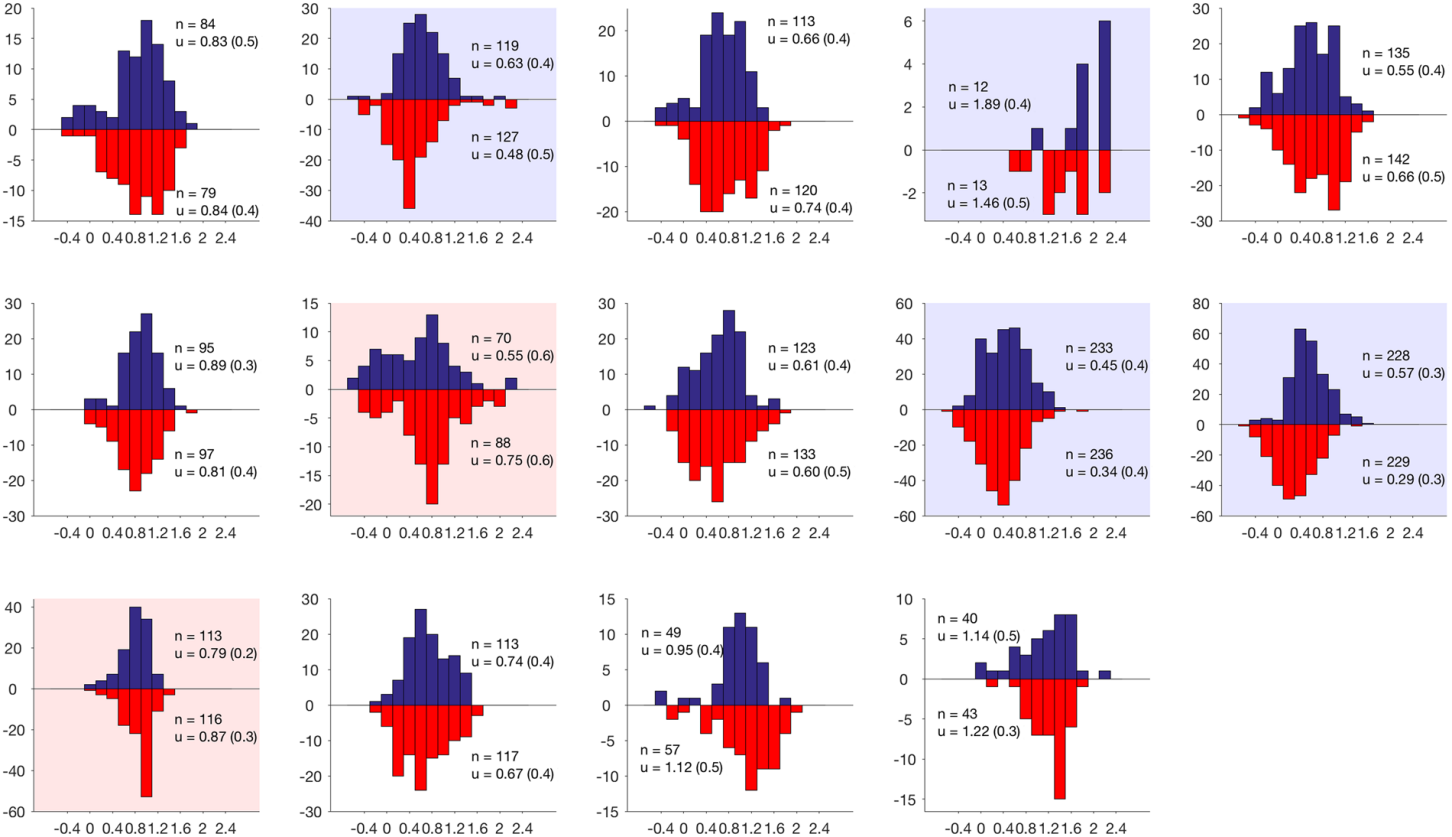
Individual level distributions for the frequency of percept (y-axis) versus log duration of percept (x-axis). Percept durations are presented logged to account for significant inter-subject variability, normalising the data. Duration histograms are shown for both percepts on the same plot. Perceived gallop rhythm identified by red bars and segregated by blue. Significant within-subject effects are shown by colouring the background for the longer duration percept (red for gallop, blue for segregated). n# refers to the number of perceptual switches and μ refers to the mean (standard deviation).

Participants reported bistable perception with 52% of the time declared gallop and 48% declared segregated based on six five-minute (500 trial) blocks, totaling 30 minutes (standard deviation across participants 8%; Fig. 2). Reports followed the typical pattern for bistable stimuli with percept duration showing a skewed distribution, which could be normalized with a log transformation (20/28 distributions non-normal at p < 0.05 according to Kolmogorov-Smirnov test for normality prior to transformation; 1/28 distributions non-normal after transformation). As is typically the case, there was substantial individual variation in mean/median percept duration (median durations ranged from 1.9–98.9 s across the 28 distributions). For each participant, the average percept duration was calculated for gallop and segregated reports and the difference between these average durations calculated. These differences were then tested across subjects for their deviation from zero using a one-sample T-test. This was tested using both mean and median as the within-subject measure of the average and prior to and after log-transformation. Whichever way the data were assessed, there was no overall statistically significant deviation from the null hypothesis of zero difference between percept durations across subjects (all Ts < 1.1, all p > 0.3), although individual participants sometimes showed significant differences (Fig. 2).

To investigate the neural correlates of auditory experience we employed canonical variate analysis^26,27^ (CVA) to identify differences between the alternative percepts without any spatial constraint. CVA models multivariate dependencies between a set of class labels (e.g. perception of gallop or segregated tones) and data features (e.g. time series of MEG data across sensors and sources). It uses the generalised eigenvalue (canonical value) solution to the treatment and residual sum of squares and products of a general linear model^27^. After transformation, the canonical values have a chi-squared distribution^27^. We report the first p-value which is formally identical to that obtained using Wilks’ Lambda and tests for the significance of any linear mapping between the stimulus condition and the sensor features^27^. Our aim was to maximize our ability to distinguish the percepts electrophysiologically at the individual participant level. The evoked response sensor level data for both perceptual states (combined) was decomposed into 5 dominant spatial modes using singular value decomposition and subsequently into 5 dominant temporal modes (also using SVD). Using the resulting 25 features we used CVA^26^ to test whether we could distinguish between the two perceptual states. To avoid biasing CVA to one trial type or the other, an equal number of trials were selected for each participant by limiting the trial count for data analysed that was associated with the more frequent percept to that of the less frequent, spreading the trial selection over the entire data acquisition. We verified that selection of equal number of trials in sequence (thus excluding some trials from the less frequent trial type later in the MEG data) did not alter the results qualitatively. Sensor space analysis using CVA could successfully distinguish the two perceptual states in 9/14 participants (64%, Table 1). We employed a linear support vector machine (SVM) analysis to classify trials over the same 25 features, running the analysis with 10-fold cross-validation over 100 iterations. The binomial test was used to identify if classification occurred at above chance level within a given subject comparing the mean correct classification rate over all trials (derived from SVM) against chance classification performance for each trial. A median 53.6% (range 50.1% to 56.4%) correct classification of trials occurred across subjects with 9/14 significant on binomial test (Table 2). Next we tested whether a non-linear SVM may improve this classification performance, using this approach we identified that 14/14 subjects could be classified above chance with this approach (median: 53.8%, range 51.8 to 56.7%), though the reader should note that the classification of trials remained weak (median: 53.8%, range 51.8 to 56.7%; Table 1 and Supplementary Table 1).

**Table 1:** Canonical Variate Analysis and Support Vector Machine classification performance of perceptual experience.

**Data type**	**MNI seed coordinates**	**CVA discrimination (%)**	**Linear SVM**		**Non-linear SVM**	
			Subject Classification (%)	Trial classification [median % (range)]	Subject classification (%)	Trial classification [median % (range)]
Sensor	N/A	9 (64)	9 (64)	53.6 (50.1–56.4)	14 (100)	53.8 (51.8–56.7)
Source	N/A	10 (71)	9 (64)	52.6 (50.3–58.3)	13 (93)	53.7 (50.4–58.1)
Right auditory cortex	54 – 14 11	11 (79)	11 (79)	54.1 (50.2–58.5)	12 (86)	54.2 (50.9–58.3)
Left auditory cortex	−49 – 20 5	7 (50)	7 (50)	51.9 (47.6–56.1)	9 (64)	52.3 (49.7–56.4)
Right posterior inferior parietal sulcus	34 – 72 38	0 (0)	0 (0)	49.5 (47.9–51.1)	6 (43)	51.4 (49.7–55.2)

Table lists the classification performance for the canonical variate analysis, linear and non-linear Support Vector Machine classification employed. Data are reported as number of subjects classified out of 14 total (%), and for SVM the median (%) and range (%) of correct trial classification.

**Table 2:** Significant decoding from consolidated ROIs.

ROI	N (out of 14)
R superior temporal	10
L inferior frontal/anterior operculum	8
R rolandic operculum/insula	7
Medial anterior frontal (bilateral)	6
L inferior temporal	6
R inferior temporal	5
L middle temporal	5
L rolandic operculum/insula	5

**Figure 3:**
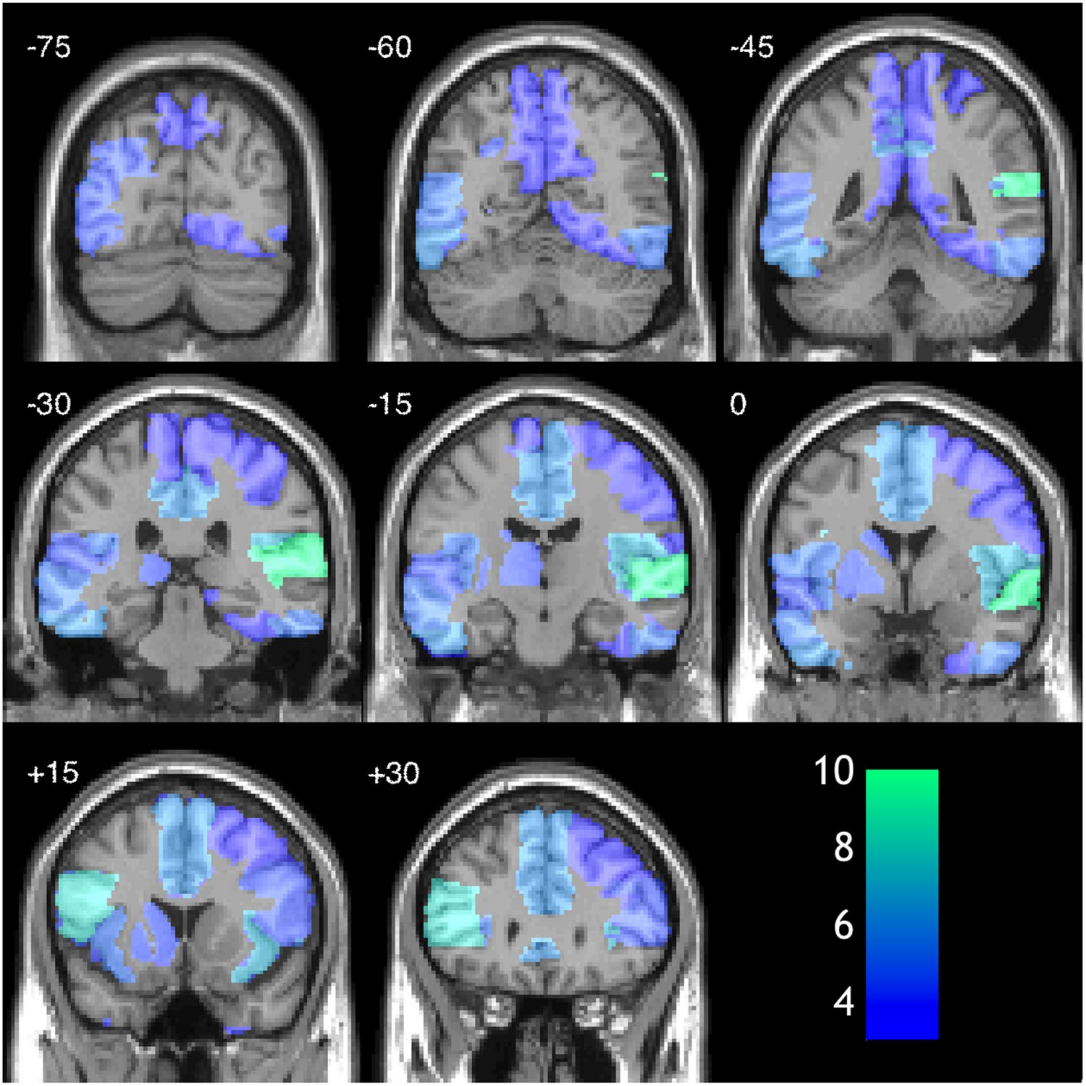
Heat map showing number of participants for whom significant differences found when data analysis was restricted to the source showing maximum posterior variance within each of 34 anatomical ROIs. The most successful region for determining differences in perceptual state was the right superior temporal cortex. Scale bar shows the number of subjects showing significant effects with analysis restricted to that ROI.

Table lists consolidated ROIs from which electrophysiological correlates of gallop and segregated streams could be discriminated in more than 5 participants (p < 0.05 at the single subject level) with canonical variate analysis. Data are graphically represented in Fig. 3.

Related analyses were then conducted in source space following reconstruction using the standard Nolte Single Shell head^28^ model in SPM12 and minimum norm inversion^29^ with 16 temporal modes. Initially we undertook an unconstrained source space analysis using CVA based on the five sources of maximal posterior variance for each participant and further collapsing across the timecourse for each source using SVD resulting in 25 spatio-temporal features. Significant electrophysiological differences between the percepts were identified in 10/14 participants (71%) with CVA (Table 1). For exploratory/illustrative purposes, the same CVA approach was used within each of 34 ROIs generated by concatenation of regions from the Automated Anatomical Labelling atlas (Fig. 3; Table 2). The region from which perception was most readily decoded was the right superior temporal cortex (10/14 (71%) of participants). Linear SVM classified an average of 52.6% (range 50.3% to 58.1%; Table 1 and Supplementary Table 1) of trials correctly, based on this unconstrained source space analysis with 9/14 subjects showing significant differences (Table 1). Non-linear SVM classified 53.7% (range 50.4 to 58.1%; Table 1 and Supplementary Table 1) of trials correctly with 13/14 subjects classified significantly above chance (p < 0.05).

Next, we conducted a source space analysis restricted to the *a priori* regions of interest (ROIs) that have been previously identified in the literature: left and right auditory cortex^1,9^ (lAC and rAC) and the right posterior intraparietal sulcus (rPIPS)^22^. Electrophysiological discrimination of the perceptual effects was possible in the right auditory cortex (CVA 11/14 subjects, linear SVM 11/14 subjects and non-linear SVM in 12/14 subjects; Table 1) and left auditory cortex (CVA 7/14 subjects, SVM 7/14 subjects and non-linear SVM 9/14 subjects; Table 1). In the right posterior intraparietal sulcus, classification was not possible with CVA or linear SVM (0/14 subjects for both) but was possible with non-linear SVM in 7/14 subjects (Table 1). The trial classification for each individual is displayed in Supplementary Table 1.

**Figure 4:**
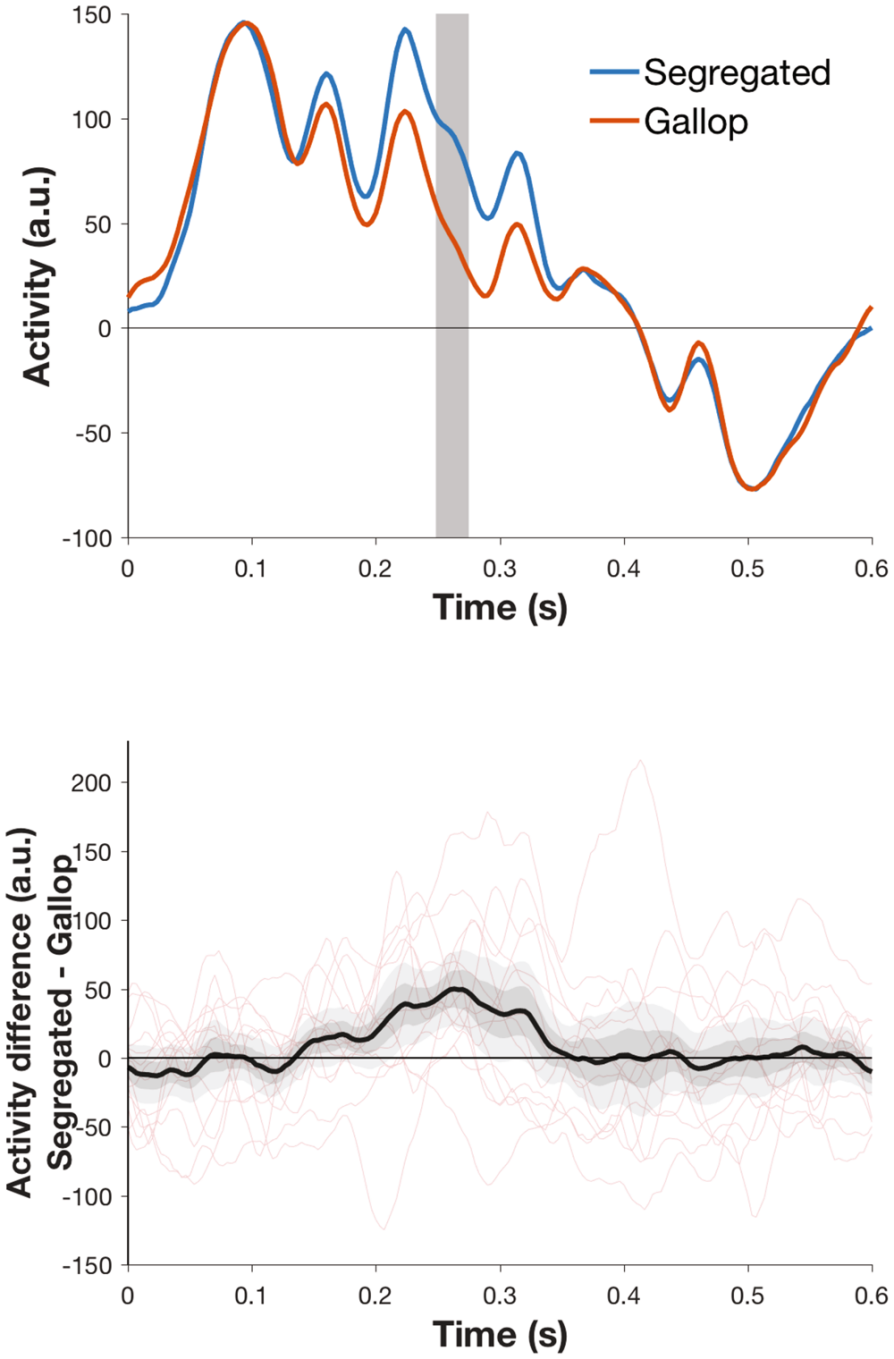
Time courses of first principal component of activity across right temporal sensors for segregated and gallop percepts (**A**). Time 0 represents onset of A tone at the start of the triplet. Grey region indicates the time window of statistical significance (250–273 ms; cluster level corrected in SPM at p < 0.05 FWE with uncorrected threshold of p < 0.005) (**B**) Activity difference-time plot showing the mean group difference in evoked response in black with + /−1 SEM in dark grey, + /−2 SEM in light grey. Individual difference plots are shown in red.

Given that we confirmed that MEG has superior ability to detect electrophysiological differences from right versus left auditory cortex^30^, we display time courses for a group level analysis informed by Gutschalk *et al*.’s previous approach^9^. Following preprocessing, trials were epoched from beginning of the first A tone to the end of the triplet sequence (0 to 600ms). Data were concatenated for each participant, baseline-corrected and underwent robust averaging in SPM. Principal components analysis (PCA) was performed over averaged evoked responses for right temporal sensors and the first principal component extracted for each participant for gallop and segregated states. Similar to the work of Gutschalk *et al*.^9^, we found that the two perceptual states could be discriminated at 250–267ms (Fig. 4, Family Wise Error corrected p < 0.05).

## Discussion

Here we replicated that a previously described behavioral paradigm for auditory bistability produces distinct perceptual states that can be distinguished electrophysiologically using MEG. At both sensor and source level, this could be achieved in the majority of participants. While classification was possible in the majority of subjects, classification of individual trials was weak, with less than 60% of trials classified correctly in any subject. This may also relate to the subjective nature of the task and noise, or heterogeneity in reporting the experience. Nonetheless, significant classification of perceptual experience was achieved, particularly with the non-linear SVM classification approach. This latter technique may have particular utility for classifying perceptual experience, at least from auditory sources.

Our study was stimulated by the controversy in the literature over the neural correlates of auditory bistability and adds weight to previous claims that the perceptual states can be distinguished electrophysiologically in the auditory cortex. Our unconstrained source space analysis identified sources predominantly within the temporal lobe supporting our region of interest approach. Interestingly our data are consistent with earlier studies demonstrating easier decoding from the right, rather than, left AC^30^. This has been attributed to the folding of the left AC making source reconstruction of signals difficult. Our data highlight that of all the regions of interest studied, the rAC is the most promising target for auditory decoding studies. However our analysis only provides limited support the previous studies by Cusack^22^ and Hill *et al*.^20^ that suggested that the rPIPS is involved in the perception of bistable auditory stimuli. We were only able to classify trials above chance using non-linear SVM analysis and then only in six subjects (Table 1 and Supplementary Table 1) from the rPIPS. However it should be noted that through our experimental design we cannot exclude that this brain region has a distinct role in the selection of the perceptual experience^23^. However rPIPS and other parietal sources were not frequently identified as amongst the sources of maximal posterior variance. Rather various temporal and frontal sources appeared to show maximal variance and improved discrimination between percepts (Fig. 3).

It has recently been demonstrated that multivariate pattern analysis can distinguish perceptual states from steady state bistable visual stimuli^5^ and during REM sleep^31^. In our study, trial wise classification was feasible at above chance levels in most subjects particularly when using a non-linear SVM classification (Table 1 and Supplementary Table 1). Hence “within subject” classification of an individual’s conscious state (with their own training data) was possible in most subjects from even a single source. However the trial classification rate was still low overall (generally less than 60%) meaning that this is not a robust effect. Given this low classification rate, we did not attempt to “cross subject” classification (with training data from other subjects). We speculate that there may be interindividual differences in the neurobiology underlying perception, and the reporting of the perception, that contribute to the poor classification rates, though we also highlight that temporal regions showed consistent effects across the two perceptual states (Fig. 4). In addition to inter-individual differences in the auditory processing of the stimulus, variations in volitional control of the percept and attention to the percept may have also contributed to the heterogeneity we observed. In order for our paradigm to be robust enough for transition into study altered states of consciousness, as we planned to do, we specifically did not attempt to control these variables.

Our future analyses will focus on within-subject decoding of perceptual experiences using time-frequency analyses looking for variance in induced activity that discriminate these states^5^. Furthermore Gutschalk and Dykstra^1^ highlight that current data do not implicate bottom-up or top-down mechanisms of stream segregation, and there has been insufficient investigation of the influence of other frontoparietal regions on the electrophysiological basis for the perceptual states. Based on the regions identified in our study (Fig. 3, Table 2), we speculate that frontotemporal nodes, and potentially their hierarchical interactions may be important determinants of bistable auditory experiences. Studies of volitional control and perceptual switches of the paradigm may help with this and would provide an interesting contrast with our study. Furthermore it would be interesting if techniques such as transcranial magnetic stimulation can alter perceptual switches or content through targeting different levels of cortical hierarchy^23,32^.

## Conclusions

Magnetoencephalography can be used to objectively classify subjective perceptual experience during auditory bistability and inform the neural correlates of consciousness for auditory experience. While trialwise classification was feasible at above chance levels, it was relatively weak though non-linear SVM approaches to classification appear promising.

## Methods

Fourteen adult participants gave informed consent to take part in the study which was approved by the UCL Graduate School Research Ethics Committee. We confirm that all experiments were performed in accordance with relevant guidelines and regulations. Auditory stimuli were generated in MATLAB and the experiment controlled with the Cogent 2000 toolbox (www.vislab.ucl.ac.uk/cogent.php). Stimuli were delivered through a piezoelectric device connected via plastic tubing to ear-inserts to the participant in the MEG machine delivered to both ears and adjusted to a comfortable range for each participant. The MEG scanner was a 275-channel CTF Omega whole-head gradiometer (VSM MedTech, Coquitlam, BC, Canada) housed in a magnetically shielded room. Data acquisition used a 600-Hz sampling rate. After participants were seated in the MEG, head localizer coils were attached to the nasion and 1 cm anterior to the left and right tragus to monitor head movement during recording.

Participants listened to the stimulus in six five-minute (500 trial) blocks (total 30 minutes) in the MEG scanner with breaks between blocks for participants’ comfort. Perceptual state was reported by participants holding one of two buttons on an MEG-compatible button box with their right hand. A projector connected to the stimulus PC controlling the experiment displayed a cross hair at the centre of the screen and text reminding participants of the button assignments throughout the block. Buttons were switched on each block and the initial button definition was counterbalanced across participants. Button presses were recorded on the stimulus PC and directly into the MEG console, and the analogue auditory stream was also recorded on the MEG console and used to check stimulus timing. Trials during which no specific percept was reported or button presses were ambiguous (e.g. both buttons held down) were omitted from analysis.

Behavioral reports of perceptual state were used to distinguish the gallop rhythm and the segregated streams of tones. As changes in perception will typically precede the motor response, we excluded trials immediately occurring before and after a perceptual switch signaled by the button press. Data were analyzed using SPM12 (http://www.fil.ion.ucl.ac.uk/spm) with standard preprocessing steps including downsampling to 300 Hz, low pass filtering at 30 Hz and high-pass filtering at 0.1 Hz with fifth order Butterworth filters. Trials were epoched from beginning of the A tone to the end of the triplet sequence (0 to 600ms). Data were concatenated for each subject, baseline-corrected and averaged using robust averaging. Robust averaging is a more sophisticated version of normal averaging, where each timepoint in each trial is weighted according to how different it is from the median across trials^33,34^. Note that while we attempted to discriminate the electrophysiological state underlying each percept as epoched from the start of each triplet, the stimulus was played continuously and hence the classical evoked response timings may be influenced by neural processing related to the immediately previous stimulus (e.g. 50ms after the first A tone in a triplet is also precisely 350ms after the last A tone from the previous triplet). Initially an unconstrained approach was adopted in which the five spatial modes (based upon sensors) or individual sources of maximum posterior variance across all trials were selected for each participant. Reconstructed time courses from these five spatial modes or sources were further reduced by taking the first five components from SVD to generate 25 data features which were then used for CVA and SVM.

SVM was implemented in MATLAB 2016a using standard methods (fitcsvm.m)^35^ and 10-fold cross validation run 100 times. The reported percentage classification rates are the mean classification accuracy across the 100 repetitions for the untrained trials. Binomial testing used the binocdf.m function in the MATLAB Stats toolbox. For non-linear SVM, the radial basis function was adopted and an initial two stage grid search was used to optimize the kernel scale and box constraint parameters for each participant individually. For the grid search, a single 10-fold classification was performed at each possible point on a 9 × 9 grid of parameter values equally spaced logarithmically for each parameter. At the first stage of the search, kernel scale was tested between 10^–3^ and 10^15^ and box constraint between 10^–3^ and 10^9^. For the second stage, a further search on a 9 × 9 grid centered at the best parameter pair + /−1 unit on the initial grid was used. The best resulting pair of parameter values from this second stage grid search was then used for 100 repetitions of 10-fold cross validation.

Source reconstruction used the standard Nolte Single Shell head^28^ model in SPM12 and employed minimum norm inversion^29^ with 16 temporal modes. Sources had to be a minimum of 15 mm apart to be considered separate. In source space, a second approach used *a priori* determined regions of interest (ROI) based upon spheres centered on left and right auditory cortex^1,9^ (lAC and rAC) and the right posterior intraparietal sulcus (rPIPS)^22^. For both these approaches the sparsely spatially sampled source time series were summarized using SVD and differences between the two perceptual states tested using the same approach outlined above for the unconstrained source/sensor level analysis (however only one source was included rather than the multiple sources used in those analyses). Finally, for illustrative purposes, we performed a whole brain analysis using 34 concatenated ROIs from the Automated Anatomical Labelling (AAL) atlas (supplementary details of ROI concatenation are available from the authors). For each subject, the maximal source of posterior variance was used for analysis within each ROI and the same analysis approach for the single ROIs outlined above was applied. The number of subjects showing significant electrophysiological discrimination within each ROI was recorded and displayed.

An additional sensor space analysis involved selection of electrodes over right temporal cortex and group-level analysis. Data were initially concatenated for each participant, baseline-corrected and underwent robust averaging in SPM. Principal components analysis (PCA) was performed over averaged evoked responses for right temporal sensors and the first principal component extracted for each participant for gallop and segregated states. The differences between the mean activity under the two perceptual states was calculated and used for statistical analysis.

### Statistical Analyses

P-values were considered significant at p < 0.05 with family-wise error correction for multiple comparisons where appropriate. Almost all analyses of MEG data described resulted in a single statistical test from CVA or SVM after collapsing across spatial and temporal dimensions and no correction for multiple comparisons was applied. The sensor space analysis derived from right temporal sensors resulted in a time course of 180 data points for each participant representing the difference between mean activity under the two perceptual states. The consistency of this time course across participants was tested using a random effects one-sample t-test in SPM12 with FWE cluster-level correction for multiple comparisons applied (uncorrected p-value of 0.005).

## Electronic supplementary material

Supplementary Table 1

## References

[1] Gutschalk A, Dykstra AR (2014). Functional imaging of auditory scene analysis. Hearing research.

[2] Frith C, Perry R, Lumer E (1999). The neural correlates of conscious experience: an experimental framework. Trends Cogn Sci.

[3] Blake RR, Fox R, McIntyre C (1971). Stochastic properties of stabilized-image binocular rivalry alternations. Journal of experimental psychology.

[4] Haynes JD, Deichmann R, Rees G (2005). Eye-specific effects of binocular rivalry in the human lateral geniculate nucleus. Nature.

[5] Sandberg K (2013). Early visual responses predict conscious face perception within and between subjects during binocular rivalry. J Cogn Neurosci.

[6] Sandberg K (2014). Distinct MEG correlates of conscious experience, perceptual reversals and stabilization during binocular rivalry. Neuroimage.

[7] van Noorden LP (1977). Minimun differences of level and frequency for perceptual fission of tone sequences ABAB. The Journal of the Acoustical Society of America.

[8] Bregman AS, Campbell J (1971). Primary auditory stream segregation and perception of order in rapid sequences of tones. Journal of experimental psychology.

[9] Gutschalk A (2005). Neuromagnetic correlates of streaming in human auditory cortex. J Neurosci.

[10] Gutschalk A, Oxenham AJ, Micheyl C, Wilson EC, Melcher JR (2007). Human cortical activity during streaming without spectral cues suggests a general neural substrate for auditory stream segregation. J Neurosci.

[11] Micheyl C, Tian B, Carlyon RP, Rauschecker JP (2005). Perceptual organization of tone sequences in the auditory cortex of awake macaques. Neuron.

[12] Pressnitzer D, Sayles M, Micheyl C, Winter IM (2008). Perceptual organization of sound begins in the auditory periphery. Curr Biol.

[13] Winkler I, Takegata R, Sussman E (2005). Event-related brain potentials reveal multiple stages in the perceptual organization of sound. Brain Res Cogn Brain Res.

[14] Kondo HM, Kashino M (2009). Involvement of the thalamocortical loop in the spontaneous switching of percepts in auditory streaming. J Neurosci.

[15] Kondo HM (2012). Separability and commonality of auditory and visual bistable perception. Cereb Cortex.

[16] Yamagishi S, Otsuka S, Furukawa S, Kashino M (2016). Subcortical correlates of auditory perceptual organization in humans. Hearing research.

[17] Hill KT, Bishop CW, Miller LM (2012). Auditory grouping mechanisms reflect a sound’s relative position in a sequence. Frontiers in human neuroscience.

[18] Snyder JS, Alain C, Picton TW (2006). Effects of attention on neuroelectric correlates of auditory stream segregation. J Cogn Neurosci.

[19] Szalardy O, Bohm TM, Bendixen A, Winkler I (2013). Event-related potential correlates of sound organization: early sensory and late cognitive effects. Biological psychology.

[20] Hill KT, Bishop CW, Yadav D, Miller LM (2011). Pattern of BOLD signal in auditory cortex relates acoustic response to perceptual streaming. BMC Neurosci.

[21] Dykstra AR (2011). Widespread Brain Areas Engaged during a Classical Auditory Streaming Task Revealed by Intracranial EEG. Frontiers in human neuroscience.

[22] Cusack R (2005). The intraparietal sulcus and perceptual organization. J Cogn Neurosci.

[23] Zaretskaya N, Thielscher A, Logothetis NK, Bartels A (2010). Disrupting parietal function prolongs dominance durations in binocular rivalry. Curr Biol.

[24] Leopold DA, Logothetis NK (1996). Activity changes in early visual cortex reflect monkeys’ percepts during binocular rivalry. Nature.

[25] Leopold DA, Logothetis NK (1999). Multistable phenomena: changing views in perception. Trends Cogn Sci.

[26] Friston KJ (1996). A multivariate analysis of evoked responses in EEG and MEG data. Neuroimage.

[27] Jafarpour A, Barnes G, Fuentemilla L, Duzel E, Penny WD (2013). Population level inference for multivariate MEG analysis. PLoS One.

[28] Nolte G (2003). The magnetic lead field theorem in the quasi-static approximation and its use for magnetoencephalography forward calculation in realistic volume conductors. Physics in medicine and biology.

[29] Friston K (2008). Multiple sparse priors for the M/EEG inverse problem. Neuroimage.

[30] Shaw ME, Hamalainen MS, Gutschalk A (2013). How anatomical asymmetry of human auditory cortex can lead to a rightward bias in auditory evoked fields. Neuroimage.

[31] Horikawa T, Tamaki M, Miyawaki Y, Kamitani Y (2013). Neural decoding of visual imagery during sleep. Science.

[32] Megumi F, Bahrami B, Kanai R, Rees G (2015). Brain activity dynamics in human parietal regions during spontaneous switches in bistable perception. Neuroimage.

[33] Wager TD, Keller MC, Lacey SC, Jonides J (2005). Increased sensitivity in neuroimaging analyses using robust regression. Neuroimage.

[34] Litvak V (2011). EEG and MEG data analysis in SPM8. Computational intelligence and neuroscience.

[35] Hsu, C. W., Chang, C. C. & Lin, C. J. *A Practical Guide to Support Vector Classification*, https://www.csie.ntu.edu.tw/~cjlin/papers/guide/guide.pdf (2016).

