# Supplementary material for: Magnetoencephalographic Correlates of Perceptual State During Auditory Bistability
Source: Sci Rep. 2018 Jan 17;8:976. doi: 10.1038/s41598-018-19287-0 (PMC5772671; doi:10.1038/s41598-018-19287-0)
Supplement: Supplementary file 1 — Supplementary Table 1 [file 41598_2018_19287_MOESM1_ESM.pdf]

# Magnetoencephalographic Correlates of

# Perceptual State During Auditory Bistability

Robert D. Sanders[1,2*]

Joel S. Winston[1,3]

Gareth R. Barnes[3]

Geraint Rees[1, 3]

RDS and JSW contributed equally to this work.

1. Institute of Cognitive Neuroscience, University College London, Alexandra House, 17-19 Queen Square, London, WC1N 3AR, London, United Kingdom
2. Department of Anesthesiology, University of Wisconsin, Madison, USA
3. Wellcome Trust Centre for Neuroimaging, University College London, London, WC1N 3BG, United Kingdom

*Correspondence to: Dr Robert D Sanders, Department of Anesthesiology, University of Wisconsin, Madison, USA. Email: robert.sanders@wisc.edu

**Supplementary Table 1:** *Individual participant trial classification by analysis type.*

Cells show SVM classification rates for individual participants for each analysis expressed as mean across 100 iterations of 10-fold cross validation (95% confidence interval across iterations is in parentheses). Pink cells show classification rates at above chance level. The far right column (in grey) shows the median classification rate across participants. ROI = region of interest, rAC = right auditory cortex, lAC = left auditory cortex, rPIPS = right posterior inferior parietal sulcus.

| Participant | | | | S01 | S02 | S03 | S04 | S05 | S06 | S07 | S08 | S09 | S10 | S11 | S12 | S13 | S14 | Median |
|---|---|---|---|---|---|---|---|---|---|---|---|---|---|---|---|---|---|---|
| Number of trials | | | | 2476 | 2022 | 1996 | 1956 | 1952 | 2502 | 2246 | 2010 | 1746 | 1206 | 2188 | 2470 | 1814 | 2756 | 2016 |
| Analysis | Sensor | | Linear | 51.2 (51.1-51.3) | 53.9 (53.8-54.0) | 51.8 (51.6-51.9) | 51.3 (51.2-51.4) | 54.4 (54.3-54.5) | 55.5 (55.4-55.6) | 53.6 (53.5-53.7) | 50.1 (50.0-50.3) | 51.6 (51.4-51.7) | 53.4 (53.3-53.6) | 54.4 (54.3-54.5) | 52.7 (52.6-52.8) | 53.7 (53.6-53.8) | 56.3 (56.3-56.4) | 53.6 |
| | Sensor | | Nonlinear | 51.7 (51.6-51.9) | 55.7 (55.6-55.8) | 52.4 (52.3-52.5) | 53.2 (53.0-53.4) | 55.2 (55.1-55.3) | 56.7 (56.6-56.8) | 54.5 (54.4-54.6) | 52.8 (52.6-52.9) | 52.0 (51.8-52.1) | 53.6 (53.4-53.8) | 54.9 (54.8-55.0) | 52.7 (52.6-52.8) | 53.9 (53.8-54.1) | 56.7 (56.6-56.8) | 53.8 |
| | Source | | Linear | 50.7 (50.6-50.8) | 51.0 (50.8-51.1) | 52.6 (52.4-52.7) | 52.5 (52.4-52.6) | 53.2 (53.0-53.3) | 54.2 (54.1-54.3) | 51.5 (51.4-51.6) | 50.3 (50.2-50.5) | 56.1 (56.0-56.2) | 58.1 (57.9-58.2) | 53.1 (53.0-53.2) | 53.4 (53.3-53.5) | 51.7 (51.5-51.8) | 52.3 (52.2-52.4) | 52.6 |
| | Source | | Nonlinear | 50.4 (50.2-50.5) | 55.2 (55.1-55.3) | 54.6 (54.5-54.7) | 52.9 (52.8-53.0) | 53.1 (53.0-53.3) | 54.3 (54.2-54.4) | 51.9 (51.8-52.0) | 53.3 (53.1-53.4) | 56.3 (56.2-56.4) | 58.1 (58.0-58.2) | 54.6 (54.5-54.7) | 53.6 (53.5-53.7) | 53.8 (53.7-53.9) | 52.6 (52.5-52.7) | 53.7 |
| | ROI | rAC | Linear | 52.6 (52.5-52.7) | 55.3 (55.2-55.4) | 54.9 (54.8-54.9) | 50.3 (50.2-50.4) | 57.1 (57.0-57.2) | 55.4 (55.3-55.4) | 50.0 (49.9-50.2) | 51.5 (51.4-51.6) | 58.4 (58.4-58.5) | 55.7 (55.6-55.8) | 53.1 (53.1-53.2) | 52.7 (52.6-52.8) | 53.4 (53.3-53.5) | 55.3 (55.3-55.4) | 54.1 |
| | ROI | rAC | Nonlinear | 52.7 (52.7-52.8) | 57.2 (57.1-57.2) | 54.8 (54.7-54.9) | 50.9 (50.7-51.1) | 57.8 (57.7-57.8) | 56.4 (56.4-56.5) | 53.0 (52.9-53.1) | 51.5 (51.3-51.6) | 58.3 (58.2-58.4) | 55.6 (55.5-55.7) | 53.6 (53.5-53.6) | 52.7 (52.7-52.8) | 53.6 (53.5-53.7) | 55.3 (55.3-55.4) | 54.2 |
| | ROI | lAC | Linear | 52.1 (52.0-52.2) | 50.4 (50.3-50.6) | 51.7 (51.6-51.8) | 50.6 (50.5-50.7) | 47.6 (47.5-47.8) | 54.4 (54.4-54.5) | 54.4 (54.3-54.4) | 51.0 (50.9-51.2) | 49.5 (49.4-49.7) | 54.1 (54.0-54.2) | 48.2 (48.1-48.4) | 54.3 (54.3-54.4) | 54.7 (54.6-54.8) | 56.1 (56.0-56.1) | 51.9 |
| | ROI | lAC | Nonlinear | 52.3 (52.2-52.4) | 50.9 (50.7-51.0) | 51.9 (51.7-52.0) | 50.8 (50.7-51.0) | 51.4 (51.3-51.6) | 54.3 (54.2-54.4) | 54.3 (54.2-54.3) | 51.0 (50.8-51.1) | 52.2 (52.1-52.3) | 54.0 (53.9-54.2) | 49.7 (49.6-49.8) | 54.8 (54.7-54.8) | 55.2 (55.1-55.3) | 56.4 (56.4-56.5) | 52.3 |
| | ROI | rPIPS | Linear | 48.0 (47.9-48.2) | 50.1 (49.9-50.2) | 48.2 (48.1-48.4) | 48.2 (48.1-48.4) | 49.8 (49.6-49.9) | 50.7 (50.6-50.8) | 51.1 (51.0-51.2) | 48.8 (48.7-49.0) | 48.8 (48.7-49.0) | 49.3 (49.1-49.4) | 49.7 (49.6-49.9) | 49.9 (49.8-50.0) | 50.8 (50.7-50.9) | 47.7 (47.6-47.9) | 49.5 |
| | ROI | rPIPS | Nonlinear | 50.2 (50.0-50.4) | 55.2 (55.1-55.3) | 52.0 (51.9-52.2) | 50.1 (50.0-50.2) | 53.6 (53.5-53.7) | 49.8 (49.7-49.9) | 51.3 (51.2-51.4) | 52.1 (51.9-52.2) | 49.7 (49.5-49.9) | 53.5 (53.4-53.6) | 51.4 (51.3-51.5) | 50.7 (50.5-50.8) | 52.6 (52.4-52.7) | 50.4 (50.3-50.5) | 51.4 |
